# The Mediating Role of Stress Perception in Pathways Linking Achievement Goal Orientation and Depression in Chinese Medical Students

**DOI:** 10.3389/fpsyg.2021.614787

**Published:** 2021-02-19

**Authors:** Yan Wang, Luping Liu, Ning Ding, Honghe Li, Deliang Wen

**Affiliations:** Institute for International Health Professions Education and Research, China Medical University, Shenyang, China

**Keywords:** achievement goal orientation, stress perception, depression, cognitive evaluation, medical students

## Abstract

Mental health problems are frequent obstacles in medical students’ careers as doctors. Given that previous studies overlook the mediation of stress perception, the current study expanded previous goal orientation researches by addressing an unexplored mechanism. This study aims to examine the mediational roles of stress perception (perceived stressors and stress-related cognition) on the relationship between achievement goal orientation and depression in medical students. A total of 1,015 Chinese 2-year medical students completed a multi-section questionnaire. Hypotheses were examined by structural equation modeling. The findings suggest that performance-avoidance goal orientation and perceived stressors both demonstrated direct facilitative effects on depression, whereas stress-related cognition demonstrated direct obstructive effects on depression. Both perceived stressors and stress-related cognition mediated the relationship between achievement goal orientation and depression. The findings spark a new perspective on motivational intervention that assist students in adopting mastery-approaching strategy as well as ways of coping with stressful academic situations. Identifying students with achievement goal orientation and providing them with the appropriate supportive services may help them to manage stress and mitigate or prevent depression.

## Introduction

Worldwide, medical schools have a large challenge of creating an academic environment that motivates students to engage in rigorous learning without compromising their health. In general, compared with students of other disciplines or the general population, students in medical school experience higher levels of psychological stress (Steiner-Hofbauer and Holzinger, 2020). A systematic review indicated that the prevalence of depression among medical students was 27.2% (Hope and Henderson, 2014; Rotenstein et al., 2016). Similarly, in China, more than 20% of medical students had depression, a rate that has continued to grow over the past decade (Gao et al., 2020; Shen et al., 2020). Medical students’ depression deserves distinct attention, it may affect students’ careers in terms of difficulty concentrating, academic performance, professional development, and may cause a variety of problems, such as dropout, identity confusion, and more cynical, less empathetic in doctor-patient relationship (Dyrbye et al., 2006).

Previous studies have suggested that beliefs (cognitive vulnerabilities) and goals (to prove self-worth) contribute to depression (Street, 2002; Rothbaum et al., 2009). Moreover, the etiology for depression has also been strongly associated with deficits in motivational and incentive functions, as goal setting and goal pursuit is presumed to be a key factor in the diagnosis of major depressive disorder. Thus, goal motivation plays a critical role in the occurrence of depression (Vergara and Roberts, 2011). Early motivation research focused primarily on a physiological point of view. In recent years, numerous studies focus on goal-orientation theory. The type of students’ academic goals is a primary variable in motivational research in an educational context (Valle et al., 2003; Meece et al., 2006). A goal may be defined as a comprehensive pattern of belief, attributions, and affects/feelings that guides action (Weiner, 1985). It is considered to determine individuals’ cognitive, affective, and behavioral response to challenges, success and failure. The goal orientation theory attempts to explain the reasons behind individual behavior; why learners set goals and how they achieve them (Vandewalle et al., 2019). In a social cognitive perspective, goal-orientation theory emphasizes the ways in which the perceived goal orientation in a learning context interacts with the learning process.

Elliot’s four-factor goal-oriented model is a widely acceptable achievement goal framework. It is comprised of mastery-approach (MAP) orientation, mastery-avoidance (MAV) orientation, performance-approach (PAP) orientation, and performance-avoidance (PAV) orientation (Elliot, 1999). Individuals who hold a MAP goal orientation tend to focus on ways to effectively master knowledge and understand the content and then develop their own abilities. A MAV goal orientation focuses on avoiding incompetency and preventing the loss of mastery over a task and tends to hold negative qualities, such as fear of failure and a low sense of self-determination. While individuals who hold a performance-approach goal orientation tend to focus on how to achieve better results, adopting this type of goal often allows for the acquisition of skills in order to try to outdo others to prove their abilities and superiority, with the intent of gaining a positive evaluation from others. Individuals who hold a performance-avoiding goal orientation tend to focus on how to avoid adverse evaluations of themselves (Tuominen-Soini et al., 2008). Several studies posit that adopting a self-worth goal orientation (e.g., seeking self-validation and avoiding proof of worthlessness) may make individuals more vulnerable to depression, while pursuing learning goals (seeking personal growth and improving one’s abilities) may introduce a protective factor (Street, 2002). Therefore, we hypothesize:

Hypothesis 1: A mastery-approach (MAP) orientation may offered a protective effect on depression;

Hypothesis 2: A performance-avoidance (PAV) orientation may make individuals more vulnerable to depression.

Though we postulated that individuals’ goal-orientation could result in either vulnerable or resistant to depression, how the effect work is still unclear. To this point, research on the effects of goal-orientation strategy on depression have focused on the mediating variables of negative events (Sideridis, 2007). Recent research has demonstrated a unidirectional link between goal-directed rumination and psychological distress, especially in terms of perceived stress (Krys et al., 2020). So far, numerous evidence supports stress as a cause of depression. Stress, which affects individuals’ behaviors, communications, and efficiencies, is defined as the sum of the non-specific responses of the body to various internal or external stimuli. It can be defined as an automatic physical response to any stimulus that requires you to adjust to change. Every real or perceived threat to body triggers a cascade of stress hormones that produces physiological changes (Ursin and Eriksen, 2007).

Cognitive stress theory posits that stress is an individual’s perception of the environment and undergoes a dynamic appraisal process (Lazarus and Richard, 1966). Cognitive assessment is an intermediate variable, between the stimulus and the response. The difference in cognitive assessment is affected by the structure of stress factors, unique individual traits, the amount of resources, and belief systems, among other things. In other words, individual traits (such as motivation characteristics) play a key role in whether a stimulus becomes a stressor for an individual and how much that individual is threatened by the stress (Baumann et al., 2005). The current study adopts the concept of stress perception, which is an individual’s physiological and psychological response to the environment through the process of that individual’s dynamic cognitive evaluation and includes not only the perceived stressors, but also the stress-related cognition. It should be noted that, a recent study showed that goal-orientation strategy is not only associated with stress perception, but also seems to be a cause of it and, as such, is viewed as a threat to depression (Vandewalle et al., 2019). Perceived goal orientation strategy is characterized as a type of stressor in which the appraisal of a potential threat plays a primary role. In addition, goal-orientation theory assumes that individuals pursue different types of implicit motivational goals, accompanied by striking differences in strategy change under stressful situations. Deterioration in performance links to a helpless response pattern (the perceived inability to surmount failure), while mastery-oriented tends to focus on remedies for failure responses (Diener and Dweck, 1978; Wooley et al., 1978). Therefore, we hypothesize:

Hypothesis 3: Both perceived stress and stress related cognition play important roles in depression occurrence.

Hypothesis 4: Stress (both perceived stress and stress related cognition) plays a mediating role between goal orientation and depression.

The kind of personal goals may not only directly influence depression, but may, by means of one’s appraisal of stress, result in depression (Vandewalle et al., 2019). Although previous research has shown that both motivational and cognitive factors can play an important role in rendering depression, less is known about the role of motivational and cognitive factors in the maintenance of depression due to stressful medical learning environment. It should be noted that, while numerous studies have explored the manner of goal attainment for the depressed individual, there is a dearth of research that examines why some individuals adopt goal orientation that in a pattern that creates a vulnerability to depression.

In sum, previous findings emphasize the integration of motivational and cognitive factors, such as goal orientation and the perception of stress, in order to recognize the different pathways that may bring about or perpetuate depression, but little consideration of how they work in tandem. The current study synthesized the evidence from existing studies and aimed to examine the relationship among medical students’ achievement goal orientation, stress perception, and depression. Moreover, in our study, we need to develop a stress perception scale which includes the stressors, the subjective experience of the stressors, the coping style, and the stress result, allowing for the clear measurement of the stress of Chinese college students. As such, the current study may contribute to implications for interventions and therapy by: (1) investigating the direct effects of goal orientation strategy as well as the perceived stressor and stress-related cognition on depression and; (2) examining the indirect effect of achievement goal orientation on depression through the perceived stressor and stress-related cognition. It may conduce to alleviate depression that help individuals put right existing maladaptive goal orientation and stress perception.

## Materials and Methods

### Procedure

This study was approved by the Human Research Ethics Committee of China Medical University. The ethical principles of the Declaration of Helsinki were respected at all times (beneficence, non-maleficence, autonomy, and justice). From March to May 2014, all medical undergraduates in their second year at China Medical University (a total of 1,186) were included for the survey. The self-report scales were administered to groups of approximately 30 students at a time (after their class meeting) in a regular classroom environment by a trained assistant. Questionnaires describing the study and consent forms were distributed to medical students. Participation was completely voluntary and anonymous. The questionnaires were collected immediately upon completion. Only students who returned consent forms were enrolled in the study. The resulting of 1,015 students agreed to participate the study and completed the questionnaires, yielding a response rates of 85.58%. There were only 16 incomplete questionnaires. The missing items of each incomplete questionnaire are less than 4.

### Participants

All participants were second year medical students and ages ranged from 18 to 24 years. The mean age of the participants was 20.39 years (SD = 0.831). The sample consisted of 1,015 medical students, of whom 66.9% (*n* = 679) were women and 24.9% females. The majority were Han people, 125 students (12.3%) were Ethnic minorities. 344 students (33.9%) were student cadre. 20.9% of them (212 persons) came from single-parent family. 239 students (23.6%) came from government accredited poverty families. Approximately 22.0% (*n* = 223) had monthly household income up to ¥1,000, 26.5% (*n* = 269) had between ¥1,001 and ¥2,000, 22.8% (*n* = 231) had between ¥2,001 and ¥3,000, and 28.8% (*n* = 292) had income of more than ¥3,001. The sample characteristics are shown in Table 1.

**TABLE 1:** Descriptive characteristics of study sample (*N* = 1,015).

Variables	Total		Non-depression (CES-D score < 20)	Depression (CES-D score ≥ 20)	χ2	*P*
	*n*	%	*n* (%)	*n* (%)		
**Gender**					2.121	0.145
Female	679	66.9%	510 (68.2%)	169 (63.3%)		
Male	336	33.1%	238 (31.8%)	98 (36.7%)		
**Ethnicity**					2.229	0.135
Han	890	87.7%	649 (86.8%)	241 (90.3%)		
Minorities	125	12.3%	99 (13.2%)	26 (9.7%)		
**Student cadre**					5.443	0.020
No	671	66.1%	479 (64%)	192 (71.9%)		
Yes	344	33.9%	269 (36%)	75 (28.1%)		
**Family type**					0.842	0.359
Two-parent	803	79.1%	597 (79.8%)	206 (77.2%)		
Single-parent	212	20.9%	151 (20.2%)	61 (22.8%)		
**Family income**					4.229	0.238
≤1000	223	22.0%	162 (21.7%)	61 (22.8%)		
1001-2000	269	26.5%	189 (25.3%)	80 (30%)		
2001-3000	231	22.8%	170 (22.7%)	61 (22.8%)		
>3000	292	28.8%	227 (30.3%)	65 (24.3%)		
**Government accredited poverty**					7.345	0.007
No	776	76.5%	588 (78.6%)	188 (70.4%)		
Yes	239	23.6%	160 (21.4%)	79 (29.6%)		
**Academic performance (self-report)**					14.655	0.002
Excellent	41	4.0%	35 (4.7%)	6 (2.2%)		
Good	228	22.5%	183 (24.5%)	45 (16.9%)		
Fair	577	56.9%	420 (56.1%)	157 (58.8%)		
Poor	169	16.7%	110 (14.7%)	59 (22.1%)		

### Measures

#### Achievement Goal Orientations Scale

The achievement goal orientations scale is a 29-item self-report measure of goal orientation. The Chinese revised version of the achievement goal orientations scale was used (Liu and Guo, 2003), which is based on the Elliot model. The goal orientation scale includes four dimensions. The dimension of mastery-approach goals consists of nine items (items 1, 5, 7, 10, 14, 17, 19, 22, 25), a focus on task mastery, learning, and understanding and evaluates performance based on progress and improvement. The dimension of performance-approach goals consists of nine items (items 3, 6, 9, 12, 13, 18, 24, 26, 29), including caring about how to surpass others. The dimension of mastery-avoidance goals consists of five items (items 4, 11, 20, 23, 27), with individuals primarily being concerned about avoiding incomprehension. The dimension of performance-avoidance goals consists of six items (items 2, 8, 15, 16, 21, 28), with individuals primarily being concerned about how not to make oneself look stupid. Given a 5-point scale, response options ranged from 1 (completely disagree) to 5 (completely agree).

#### Stress Perception Scale

A standardized procedure was followed for the development of a new scale (Ntoumanis et al., 2005). According to social readjustment rating scale (Holmes and Rahe, 1967), perceived stress scale (Cohen et al., 1983), relevant stress literature in individual reports (Keller et al., 2012), we designed an interview outline for medical students. The interview contained three open questions: ‘what kind of stressors are you experiencing in your current learning and life,’ ‘what role does stress play in your daily learning and life’ and ‘how do you deal with stressors.’ In medical students’ interview, saturation occurred at a sample size 22. Fourteen items based on fore-mentioned scales and interviews were subsequently created and reviewed by three psychologists (the faculties of China Medical University) to confirm the academic relevance. The presentation of four items were modified according to the experts’ suggestion, which yielded the initial version V1.0. This version was then tested in a small sample of 248 medical students. After reliability and validity analysis, eleven items (3 items with factor loadings less than 0.4 were excluded, Supplementary Table 1) were selected as the final version V2.0. V2.0 Scale comprises eight related to the perceived stressor items and three stress-related cognition involving items. Cronbach’s alpha values, composite reliability coefficients, and average variance extracted for the scale are shown in Supplementary Table 2.

Perceived stressor sub-scale were classified into three potential stressor domains, namely academic performance pressure, self-development trends, and social stressors. Responses used the original 5-point Likert format from ‘strongly does not fit me’ to ‘strongly fit me.’ Stress-related cognition is defined as an individual’s perception of stress that affects their health and health outcomes, as well as who they ask for help. Stress-related cognition sub-scale mainly includes three items: amount of Stress, perception of stress affecting learning and life, and inquiry of help for stress reduction. Responses were modeled using the original 5-point Likert format. Exploratory factor analysis and confirmatory factor analysis were used to confirm a good measurement parameter in the present study (as shown in Tables 2,3).

**TABLE 2:** Confirmatory factor analysis for three scales.

Factor Model	χ^2^	df	χ^2^/df	*P*-Value	RMSEA	90% C.I.		CFI	TLI	SRMR
**Achievement goal orientation**									
Four first-order factors	193.54	41	4.721	<0.001	0.055	0.047	0.064	0.946	0.928	0.047
**Stress perception**									
Two first-order factors	992.54	337	2.945	<0.001	0.039	0.035	0.042	0.920	0.903	0.053
**Center for Epidemiological Studies-Depression**									
Three first-order and a second-order factors	581.12	166	3.501	<0.001	0.042	0.037	0.046	0.944	0.936	0.036

**TABLE 3:** Cronbach’s alpha values for three scales.

Scale	Cronbach’s alpha
**Achievement goal orientation**	
Total	0.83
Mastery-Approach (MAP)	0.78
Performance-Approach (PAP)	0.79
Performance-Avoidance (MAV)	0.72
Mastery-Avoidance (PAV)	0.78
**Stress perception**	
Total	0.81
Perceived Stressors (PS)	0.80
Stress Related Cognition (SRC)	0.71
**Center for Epidemiological Studies-Depression**	
Total (Depressive Symptoms, DS)	0.90
Somatic Concerns (SC)	0.83
Depressive Affect (DA)	0.82
Anhedonia (AN)	0.69

#### Center for Epidemiological Studies-Depression Scale (CES-D)

The CES-D has been considered to be a validated scale of depression (Radloff, 1977; Zhang et al., 2010). Previous studies have indicated that the CES-D scale is a valid measure for the assessment of depression in Chinese university students (Ling et al., 2016; Jiang et al., 2019). It contains 20 items with a 4-point rating scale scored “0” (rarely) to “3” (all of the time). Higher score indicates severer depressive symptoms. The current study adopted a cut-off score of 20 for detecting subthreshold depression. The CES-D has been validated in Chinese samples, for which a three-factor model was the best fit (Jiang et al., 2018). The three factors were somatic concerns (items 1, 2, 3, 5, 6, 7, 9, 18, 11), depressive affect (items 10, 13, 14, 15, 17, 19, 20), and anhedonia (items 4, 8, 12, 16).

### Control Variables

To control for potential effects of socio-demographic characteristics, the following variables were measured: participants’ gender (0 = male, 1 = female), ethnicity (0 = Han, 1 = Ethnic Minority), family type (0 = two-parent, 1 = single-parent), and family income (1 = ≤ 1000, 2 = 1001-2000, 3 = 2001-3000, and 4 ≥ 3000). The aforementioned variables are known to be related to quantitative stress perception and depression (e.g., Cheung and Bagley, 1998; Ryder et al., 2008; Park and Lee, 2020). We performed a hierarchical regression analysis in which depression symptoms was regressed on achievement goal orientation, stress perception and the control variables. The control variables were not significantly related to depression. Therefore, the control variables were excluded from further analyses.

### Data Analysis

Missing data were replaced using the median of a nearby point. Initial descriptive statistics and Pearson correlations were conducted in SPSS 25.0. Exploratory factor analysis was conducted in SPSS 25.0 and then a confirmatory factor analysis (CFA) using Mplus 8.3 was performed to test the factorial structure of each scale (Ryu, 2011). A structural equation model (SEM) was used to examine the effects of goal orientation on depression through PS and SRC (Yang et al., 2019; van Dam et al., 2020). A bootstrapping method with 1,000 samples and 95% confidence intervals (CI) was used to verify mediation effects (using Mplus 8.3). Model fit indices above a value of 0.90 for the ML-based indices (TLI, CFI), lower than 0.08 for SRMR, and lower than 0.06 for RMSEA are recommended as best practice (Hu and Bentler, 1999).

## Results

### Sample Characteristics of Depression

The sample consisted of 1,015 medical students, 24.9% females and 29.2% males were considered to have depressive symptoms (CES-D score ≥ 20). More detailed information on sample characteristics can be found in Table 1. The chi-square test demonstrated that those in a student cadre group had a lower depression rate than those in a non-student cadre group, the government accredited poverty group had higher depression rate than non-government accredited poverty group, and the good academic performance group had a lower depression rate than the poor academic performance group.

### Reliability and Validity Analysis of the Goal Orientation Scale, Stress Perception Scale, and Center for Epidemiological Studies Depression Scale (CES-D)

**FIGURE 1:**
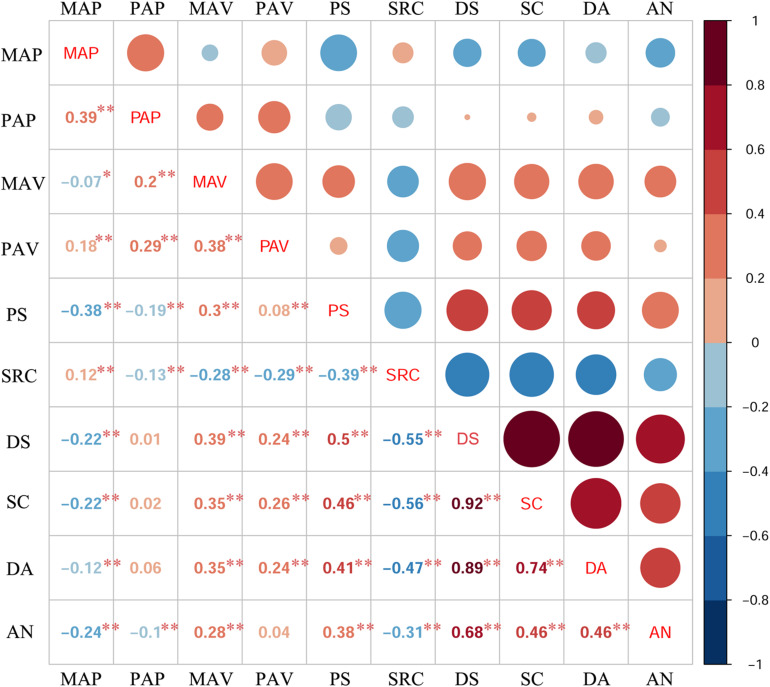
Correlations between study variables. Note: *p < 0.05, **p < 0.01.

The factorial structure of each scale was first explored by EFA and then tested via CFA. The results showed that all scales demonstrated acceptable fit indices with acceptable factor loadings (all loadings *p* < 0.001). Table 2 summarizes the model fit of statistics for the three scales. The results of the current study support a four first-order factors structure, namely mastery-approach (MAP) goal orientation, performance-approach (PAP) goal orientation, mastery-avoidance (MAV) goal orientation, and performance-avoidance (PAV) goal orientation for an achievement goal orientation scale. Furthermore, the current study lend support for a two first-order factors structure, namely perceived stressor (PS) and stress-related cognition (SRC) for the stress perception scale and a three first- and second-order factor structure, namely the second-order factor is measured by three first-order factors somatic concerns (SC), depressive affect (DA), and anhedonia (AN) for the CES-D scale. As reported in Table 3, Cronbach’s alpha demonstrated good reliability and internal consistency for the three scales. Factor loadings, composite reliability coefficients, and average variance extracted for the variables are shown in Supplementary Tables 3–6. The zero-order correlations (r-values) among study variables are presented in Figure 1.

### Description of Academic Performance Characteristics of Study Variables

As the study variables are moderate to high levels of non-normality, which may create problems in analyses, all study variables in the current study for self-report academic performance characteristics among achievement goal orientation, stress perception, and the CES-D used non-parametric tests. As shown in Table 4, a Kruskal-Wallis H test revealed that academic performance had a significant effect on the level of MAP (χ^2^ = 56.265, *p* < 0.001), PAP (χ^2^ = 40.208, *p* < 0.001), MAV (χ^2^ = 12.606, *p* < 0.001), PAV (χ^2^ = 8.673, *p* < 0.001), PS (χ^2^ = 33.875, *p* < 0.001), DS (χ^2^ = 17.897, *p* < 0.001), SC (χ^2^ = 16.156, *p* < 0.001), and AN (χ^2^ = 20.662, *p* < 0.001). Dunn’s *post hoc* multiple comparisons test revealed that, compared with the poor academic performance group, the excellent academic performance group had a higher level of MAP (*p* = 0.002) and the good academic performance group had a higher level of MAP (*p* < 0.001) and PAP (*p* < 0.001), whereas the excellent academic performance group had a lower level of MAV (*p* = 0.011), PS (*p* = 0.005), DS (*p* = 0.016), SC (*p* = 0.018), and NA (*p* = 0.006) and the good academic performance group had a lower level of PAV (*p* = 0.028), PS (*p* < 0.001), DS (*p* = 0.001), SC (*p* = 0.002), and NA (*p* = 0.001). Compared with the fair academic performance group, the excellent academic performance group had a lower level of MAV (*p* = 0.026), the good academic performance group had a lower level of PS (*p* < 0.001) and AN (*p* = 0.04), and the poor academic performance group had a lower level of MAP (*p* = 0.001), whereas the good academic performance group had a higher level of MAP (*p* < 0.001) and PAP (*p* < 0.001).

**TABLE 4:** The detail of academic performance characteristics of participants and their scale scores.

Variables	Total		Academic performance (self-report)									
			Excellent		Good		Fair		Poor		H	*P*
	Mean ± SD	*n* (missing)	Mean ± SD	*n*	Mean ± SD	*n*	Mean ± SD	*n*	Mean ± SD	*n*		
**Achievement goal orientation**												
MAP	31.01 ± 28.34	1010 (5)	32.54 ± 5.42**	41	32.90±4.46⁢**#⁢#	227	30.84 ± 5.04	574	28.65 ± 6.26^##^	168	56.265	<0.001
PAP	31.61 ± 33.12	1012 (3)	32.27 ± 6.69	41	33.69±5.29⁢**#⁢#	226	31.26 ± 5.3	576	29.87 ± 6.77	169	40.208	<0.001
MAV	13.71 ± 21.87	1013 (2)	11.56±4.03⁢**#⁢#	41	13.18 ± 4.09	228	13.9 ± 4.66	575	14.33 ± 5.39	169	12.606	0.006
PAV	16.58 ± 14.39	1013 (2)	16.20 ± 4.65	41	17.11 ± 3.77*	228	16.59 ± 3.6	575	15.94 ± 4.15	169	8.673	0.034
**Stress perception**												
PS	20.83 ± 25.58	1009 (6)	19.10 ± 4.55**	41	19.42±4.18⁢**#⁢#	226	21.10 ± 5.22	573	22.18 ± 5.20	169	33.875	<0.001
SRC	11.17 ± 4.64	1014 (1)	11.73 ± 1.70	41	11.30 ± 2.20	228	11.16 ± 2.10	576	10.91 ± 2.34	169	6.105	0.107
**CES-D (Depression)**												
DS	15.39 ± 83.47	1015 (0)	12.54 ± 7.78*	41	13.81 ± 8.32**	228	15.56 ± 9.05	577	17.6 ± 10.22	169	17.891	<0.001
SC	7.23 ± 21.00	1015 (0)	6.00 ± 4.12*	41	6.59 ± 4.17**	228	7.21 ± 4.50	577	8.47 ± 5.22	169	16.156	0.001
DA	4.66 ± 13.27	1015 (0)	3.98 ± 3.22	41	4.10 ± 3.27	228	4.78 ± 3.56	577	5.17 ± 4.34	169	7.508	0.057
AN	3.50 ± 6.03	1015 (0)	2.56 ± 1.94**	41	3.12±2.52⁢**#	228	3.57 ± 2.42	577	3.97 ± 2.49	169	20.662	<0.001

*compared with Poor group, *P* < 0.05; **compared with Poor group, *P* < 0.01; ^#^compared with Fair group, *P* < 0.05; ^##^compared with Fair group, *P* < 0.01.

### The Measurement and Structural Models

The SEM comprises a measurement model and a structural model. The model fit information of each full measurement model are presented in Table 5. All indicators are acceptable. Next, the structural model component was conducted to test whether achievement goal orientation is predictive of depressive symptoms and whether stress perception acts as a mediator in the relationship between goal orientation and depression (DS, SC, DA, or AN). Item parceling (dividing by factor loading and item content) was used and each variable had two or three packs (Jiang et al., 2018). All indices showed excellent model fit. All factor loadings for mastery-approach (MAP), performance-approach (PAP), mastery-avoidance (MAV), performance-avoidance (PAV), perceived stressors (PS), stress-related cognition (SRC), depressive symptom (DS), somatic concerns (SC), depressive affect (DA), and anhedonia (AN) were significant (*p’s* < 0.001), indicating an acceptable measurement model.

**TABLE 5:** Model fit information for the measurement model.

Model	SEM Model (Bootstrap = 1000)	χ^2^	df	χ^2^/df	*P*-Value	RMSEA	90% C.I.	CFI	TLI	SRMR
Model 1	MAP/PAP/MAV/PAV→PS/SRC→DS	827.91	228	3.63	<0.001	0.051	0.047 0.055	0.940	0.927	0.047
	MAP/PAP/MAV/PAV→PS/SRC→SC	517.53	131	3.95	<0.001	0.054	0.049 0.059	0.947	0.931	0.042
	MAP/PAP/MAV/PAV→PS/SRC→DA	473.13	131	3.61	<0.001	0.051	0.046 0.056	0.951	0.936	0.041
	MAP/PAP/MAV/PAV→PS/SRC→AN	438.46	114	3.85	<0.001	0.053	0.048 0.058	0.948	0.930	0.043
Model 2	PS/SRC→MAP/PAP/MAV/PAV→DS	827.91	228	3.63	<0.001	0.051	0.047 0.055	0.940	0.927	0.047

**FIGURE 2:**
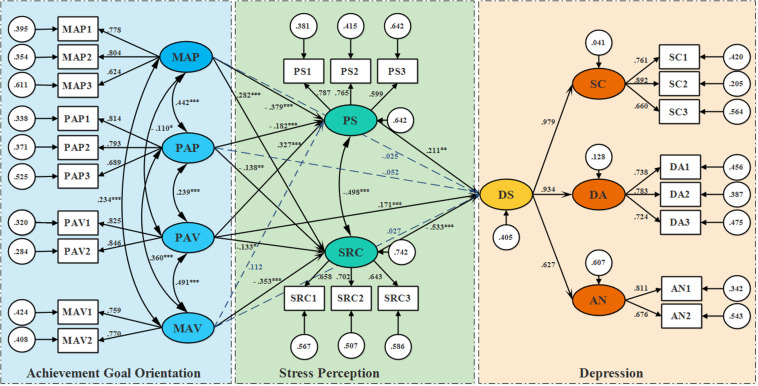
Structural model with path coefficients. Note: MAP = Mastery-approach, MAV = Mastery-avoidance, PAP = Performance-approach, PAV = Performance-avoidance, PS = perceived stressors, SRC = Stress related cognition, DS = Depressive symptoms, SC = Somatic concerns, DA = Depressive affect, AN = Anhedonia. The path coefficients are standardized. *p < 0.05, **p < 0.01, ***p < 0.001.

In model 1, achievement goal orientation was used as the predictor variable, depression as the outcome variable, and the perceived stressor (PS) and stress-related cognition (SRC) as the mediating variables to build the SEM using Mplus software. The structural model contains of direct paths from achievement goal orientation to depression and of two mediators between achievement goal orientation and depression. A visual depiction of the model (with DS as the outcome variable) is presented in Figure 2. Results (Figure 2) showed that the standardized path coefficients from mastery-approach (MAP) to perceived stressors (PS), performance-approach (PAP) to PS, performance-avoidance (PAV) to PS, MAP to stress-related cognition (SRC), PAP to SRC, mastery-avoidance (MAV) to SRC, performance-avoidance (PAV) to SRC, PAV to depressive symptom (DS), PS to DS, and SRC to DS were all significant, while MAP to DS, PAP to DS, and MAV to DS were non-significant.

The alternative model (Model 2) examined stress variables function as predictors and the four types of achievement goal orientation as the mediation variables. Comparing the two models, the model 2 showed a same fit to the Model 1 (Table 5). The two models are equivalent models. Comparing the two model indicators, we can’t rule out any model. However, form the theoretical perspective of cognitive theory of stress, goal-orientation approach act as dispositional traits affect the process from stimulus (stress) to response (emotion and/or behavior) (detailed illustration refers to discussion section). So, we retained Model 1 as the final model of the study.

### Mediation Analysis of Depression on Goal Orientation

**FIGURE 3:**
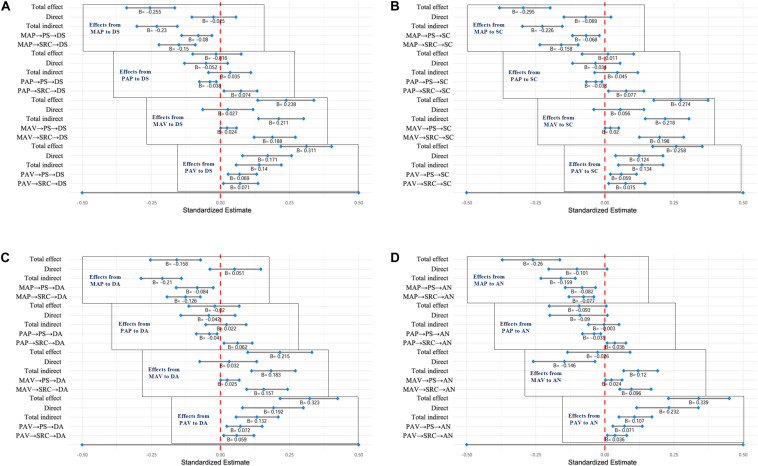
Standardized estimates of total, direct, total indirect, and specific indirect effects. **(A)** Effects from MAP, PAP, MAV, or PAV to DS; **(B)** Effects from MAP, PAP, MAV, or PAV to SC; **(C)** Effects from MAP, PAP, MAV, or PAV to DA; **(D)** Effects from MAP, PAP, MAV, or PAV to AN. If zero is not in the 95% CI, it is presumed that the effect is statistically significant at the 0.05 level.

Mediation tests were conducted by using a 1,000 bootstrapped procedure to compute 95% confidence intervals (CI). If the 95% CI for the indirect effect estimate does not include zero, it is presumed that the indirect effect is statistically significant at the 0.05 level (Shrout and Bolger, 2002; Tian et al., 2017). As shown in Figure 3A, a significant indirect path from mastery-avoidance (MAP) to depressive symptom (DS) was mediated by perceived stressors (PS) (β = −0.08, 95% CI [−0.14, −0.031]; *p* = 0.003), which accounted for 31.37% of the total effect, suggesting a buffering effect from MAP to DS. A significant indirect path from MAP to DS was mediated by stress-related cognition (SRC) (β = −0.15, 95% CI [−0.222, −0.091]; *p* < 0.001), which constituted 58.52% of the total effect, suggesting a buffering effect from MAP to DS. A significant indirect path from performance-approach (PAP) to DS was mediated by PS (β = −0.038, 95% CI [−0.076, −0.015]; *p* = 0.016), and a significant indirect path from PAP to DS was mediated by SRC (β = 0.074, 95% CI [0.013, 0.133]; *p* = 0.013), suggesting a facilitating effect from PAP to DS. A significant indirect path from mastery-avoidance (MAV) to DS was mediated by SRC (β = 0.188, 95% CI [0.122, 0.271]; *p* < 0.001), suggesting a facilitating effect from MAV to DS. A significant indirect path from performance-avoidance (PAV) to DS was mediated by PS (β = 0.069, 95% CI [0.028, 0.131]; *p* = 0.007), which constituted 22.19% of the total effect and a significant indirect path from PAV to DS was mediated by SRC (β = 0.071, 95% CI [0.011, 0.136]; *p* = 0.025), which constituted 22.83% of the total effect, suggesting a facilitating effect from PAP to DS. The results of observed total, direct, and indirect effect from MAP, PAP, MAV, and MPV to SC, DA, and AN are showed in Figures 3B–D respectively.

## Discussion

The primary findings of the current study are threefold. First, mastery-approach (MAP) orientation may offered a protective effect on depression via the mediating role of stress perception. Second, performance-avoidance goal orientation and the perceived stressor both showed direct facilitative effects on depression, whereas stress-related cognition showed direct protective effects on depression. Third, both the perceived stressor and stress related cognition may mediate the effects of goal orientation on depression.

The current research replicated and extended previous findings in various ways. To the best of our knowledge, this is the first study in medical students’ setting that incorporated mental health (i.e., depression, including depressive symptoms and the three sub-factors including somatic concerns, depressive affect, and anhedonia) and related it to achievement goal orientation and stress perception (both perceived stress and stress-related cognition). In line with our expectations (Hypothesis 3), the perceived stressor is positively related to depression and appears to have direct facilitative effects on depression. Indeed, a substantial body of research has established a robust and causal association between stressful life events and depression (Hammen, 2005). The current finding is surprising given the numerous studies exploring the relationship between perceived stressors and mental health that exist, yet there is a dearth of research examining the relationship between an individual’s perception (stress-related cognition) that stress affects their health, health outcomes, and from whom to seek help to reduce stress (Keller et al., 2012). In the current study, stress-related cognition was negatively related to depression and appeared to have direct protective effects on depression, suggesting the importance of improved stress-related cognition for optimal psychological functioning. Specifically, better stress-related cognition in the current study suggests that insight into stress perception, taking stress as a positive motivator, and relying on people close to the individual for support and helpful advice.

It should be noted that the findings on mastery-approach (MAP) goal orientation reported here did not provide support for a direct effect on depression, however, the results of the mediating effects of the perceived stressor and stress-related cognition were consistent with our expectations that MAP goal orientation offered a protective effect on depression in Chinese medical students (Hypothesis 1 and Hypothesis 4). Specifically, students with MAP goal orientation were inclined to perceived less stress, suggesting that MAP goal orientation may optimize stress as a perceived stressor by offering the advantage of providing additional information relevant to how to improve (Tuominen-Soini et al., 2008). People who use this type of goal orientation also tend to have good insight on stress perception, taking stress as a positive motivator, and relying on those closest to them for support and helpful advice, which may provide for improved mental health. Contrarily, students with mastery-avoidance (MAV) goal orientation appeared to have facilitative effects on depression and those effects were primarily mediated by stress-related cognition. In line with the definitions, mastery goal orientation tends to focus on the improvement of ability, in which MAP goal orientation implies a focus on improvement of one’s competence and gaining mastery over a task, while MAV goal orientation implies the focus is on avoiding incompetency and preventing the loss of mastery over a task. People with MAP goal orientation tend to conceptualize ability as a malleable construct that can be improved through greater effort (Elliot, 1999; Vandewalle et al., 2019). People with MAV goal orientation, however, tend to view that once one’s competencies are up to par, they will not deteriorate and often have a low perceived stress. However, this type of orientation is plausibly less adaptive in an educational setting, where the focus is on further developing one’s competencies.

Moreover, there was no direct effect on depression in students with performance-approach (PAP) goal orientation. The current results suggest that this relationship was the weakest in the model. The total mediation effects of the perceived stressor and stress-related cognition from PAP to depression were canceled out; the perceived stressor mediated the obstructive effects on depression, while stress-related cognition mediated the facilitative effects on depression. This effect was not consistent with the general supposition that PAP goal orientation is considered to be more likely be related to the perceived stressor. One possible explanation for the absence of a relationship between PAP goal orientation and the perceived stressor is that the students who utilized a performance approach may have higher self-efficacy, which may aid with the coping of stress. As previously discussed, PAP goals (demonstrating ability and outperforming others) are positively related to persistence and achievement outcomes, particularly in college students (Harackiewicz et al., 2002). Contrarily, students with performance-avoidance (PAV) goal orientation demonstrated a more direct facilitative effect on depression (in line with our expectations of Hypothesis 2) and a minute facilitative mediation effect both from perceived stressor and stress-related cognition on depression. As previous studies reveal that PAV goal orientation primarily produces adverse effects, e.g., negative cognitions, anxiety, depression, self-concerns, and distraction (Sideridis, 2007). It should be noted that, although students with performance goal orientation (both PAP and PAV) may have good insight into stress perception, taking stress as a positive motivator and relying on those close to them for support and helpful advice, those with PAP goal orientation tend to focus on showing one’s competence and gaining positive judgments from others, while students with PAV goal orientation focuses on avoiding any display of incompetence and preventing unfavorable judgments from others, which could prevent them from getting real psychological support.

Though many equivalent models can fit the data equally well, equivalent models cannot be distinguished in statistical ways (Raykov and Marcoulides, 2007). Exploration of relationships among variables without *a priori* specification may result in statistical significance but have little theoretical significance. In our study, we rule out equivalent Model 2 mainly based on the theoretical guidance of cognitive stress theory. There are three phases to this theory (Folkman and Lazarus, 1986). First, primary appraisal includes the process of identifying whether the stress poses a threat to the individual (Lazarus and Richard, 1966). In the primary appraisal process there are two factors that affect whether the individual defines the stress as a threat to himself or herself; one is the stimulus structural factor, such as the urgency of the expected injury or the ambiguity of the stimulus cues, while the other is the individual psychological structural factor (personality trait) (Lazarus and Richard, 1966; Hoyt et al., 2016), such as motivational characteristics, the belief systems about the environment, intellectual resources, educational level, etc., Next is the secondary appraisal. Once a stimulus is determined to be threatening, the individual will mentally consider an appropriate response to the stressor in order to reduce the anticipated harm. This process is influenced by three factors; the degree of the threat, the stimulus structure, and the psychological structure of the individual, including motivation type, self-resources, defensive characteristics, and belief in environment and personal resources. Third is coping, the process by which a secondary assessment is made to determine the response strategy that should be adopted and implemented. Coping strategies can include direct action, defensive re-evaluation, or an anxiety response. Goal-orientation approach as the motivational characteristics may affect the first and second process of response formation. They act as dispositional traits affect the process from stimulus (stress) to response (emotion and/or behavior).

The exploratory factor analysis results for CES-D from the current study were similar to previous studies (Jiang et al., 2019). It seems that the Chinese students may have an overlap of understanding between depressed affect and somatic concerns. Considerable studies have demonstrated that the Chinese tend to express somatization of depression, whereas Westerners tend to express the psychological symptoms of depression (Cheung and Bagley, 1998; Ryder et al., 2008). The current study showed a 26.3% prevalence of depression, which was similar to a previous meta-analysis (Puthran et al., 2016). Overall, the current study shows no significant gender difference in depression, although some research has demonstrated a higher depression rate in female students (Lloyd and Gartrell, 1984; Dyrbye et al., 2006), numerous studies found no gender difference in depression proportions at the start of medical school, but greater increases in female students through the course of training (Chen et al., 2013; Gao et al., 2020). Ethnicity information also revealed a similar depression rate among minority students and Chinese Han students, consistent with a previous study (Chen et al., 2013). Unexpectedly, students with a single-parent family showed a higher perceived stress but a similar depression rate compared with students with a two-parent family. Previous studies have shown that non-intact families generally had higher risk of mental health issues such as depression, suicidal ideation, and poor perceived health status than two-parent families (Park and Lee, 2020). One explanation for the different results is that perhaps medical students are already excellent and students who have psychological problems due to family status may have been excluded from admission to medical school. Furthermore, student cadres had lower depression rates than non-cadre students. The student cadres are the leaders of the students and are generally excellent students, have better communication skills, and overall more positive personalities, which can enhance their mental health. Typically, students with government accredited poverty families had higher depression rates than non-government accredited poverty families (Shamsuddin et al., 2013; Chan and Sun, 2020), although there was no significant difference in income on depression, perhaps because college students may not clearly know their family income, but do clearly know whether their family was a government accredited poverty family, as this status would provide eligibility for special scholarships and policy care in Chinese universities. The current study suggests possible important academic performance differences in the prediction of risk to medicals students’ depression. Specifically, students with good academic performance had lower depression rates than those with poor academic performance. For medical students, perhaps, depressive feelings of academic disappointment may be more prevalent among those who have poor academic performance, as failure in academic tasks is almost as frequent as the number of exams and evaluations in which they participate (Stewart et al., 1999; Yusoff et al., 2013).

Given the methodological limitations of the present study, there are some limitations in this study. First, this research is cross-sectional in nature and therefore makes it difficult to rule out other orders of pathways. This research represents an exploratory stage, so the next logical step is to conduct laboratory experimental researches or use longitudinal study design to properly examine the causality among those variables. Second, future research should explore key individual variables that influence the direction of stress perception, such as self-esteem or other personality traits, to better understand the development of depression in medical students. Third, more attention should be paid to the contextual factors, such as the classroom learning climate, that influence the achievement goal orientation of medical students. Finally, inferences should be derived with caution from the current research, as the effect size is variable and some effects are small. The second year students have more stable goal orientation, and they are not busy with clinical clerkship.

In summary, the current results highlight the important links between goal characteristics and their facilitative and obstructive effects on depression via the perceived stressor and stress-related cognition. The empirical findings spark a more holistic perspective on the application of motivational intervene that assist students in adopting mastery-approaching strategy as well as ways of coping with stressful academic situations. Identifying students with achievement goal orientation and providing them with the appropriate supportive services may help them to manage stress and mitigate or prevent depression.

## Data Availability Statement

The dataset supporting the conclusions of this article is included within its additional files (Supplementary Dataset).

## Ethics Statement

The studies involving human participants were reviewed and approved by Human Research Ethics Committee of China Medical University. Written informed consent to participate in this study was provided by the participants’ legal guardian/next of kin.

## Author Contributions

LL, HL, ND, and DW substantially contributed to the conception and design of the research. LL contributed to data acquisition. YW analyzed the data, interpreted the results, and prepared the initial draft of the manuscript. ND critically reviewed the manuscript and gave advice for modifications. HL, ND, and DW worked for the final approval of the version of the manuscript to be published. All authors contributed to the article and approved the submitted version.

## Conflict of Interest

The authors declare that the research was conducted in the absence of any commercial or financial relationships that could be construed as a potential conflict of interest.

## Abbreviations

AN: Anhedonia
CES-D: Center for Epidemiological Studies-Depression scale
CFA: Confirmatory factor analysis
CFI: Comparative fit index
CI: Confidence intervals
DA: Depressive affect
DS: Depressive symptoms
EFA: Exploratory factor analysis
GAS: General Adaptation syndrome
MAP: Mastery-approach
MAV: Mastery-avoidance
PAP: Performance-approach
PAV: Performance-avoidance
PS: Perceived stressor
RMSEA: Root mean square error of approximation
SC: Somatic concerns
SEM: Structural equation model
SRC: Stress-related cognition
SRMR: Standardized root mean square residual
TLI: Tucker-Lewis index.

## References

[B1] Baumann N. Kaschel R. Kuhl J. (2005). Striving for unwanted goals: stress-dependent discrepancies between explicit and implicit achievement motives reduce subjective well-being and increase psychosomatic symptoms. *J. Personal. Soc. Psychol.* 89 781–799. 10.1037/0022-3514.89.5.781 16351368

[B2] Chan H. W. Q. Sun C. F. R. (2020). Irrational beliefs, depression, anxiety, and stress among university students in Hong Kong. *J. Am. Coll Health* 2020 1–15. 10.1080/07448481.2019.1710516 32149578

[B3] Chen L. Wang L. Qiu X. H. Yang X. X. Qiao Z. X. Yang Y. J. (2013). Depression among Chinese University Students: Prevalence and Socio-Demographic Correlates. *PLoS One* 8:e58379. 10.1371/journal.pone.0058379 23516468 PMC3596366

[B4] Cheung C.-K. Bagley C. (1998). Validating an American scale in Hong Kong: the Center for Epidemiological Studies Depression Scale (CES-D). *J. Psychol.* 132 169–186. 10.1080/00223989809599157 9529665

[B5] Cohen S. Tom K. Robin M. (1983). A Global Measure of Perceived Stress. *J. Health Soc. Behav.* 24 385–396. 6668417

[B6] Diener C. I. Dweck C. S. (1978). An analysis of learned helplessness: Continuous changes in performance, strategy, and achievement cognitions following failure. *J. Personal. Soc. Psychol.* 36 451–446.

[B7] Dyrbye L. N. Thomas M. R. Shanafelt T. D. (2006). Systematic review of depression, anxiety, and other indicators of psychological distress among U.S. and Canadian medical students. *Acad. Med.* 81 354–373. 10.1097/00001888-200604000-00009 16565188

[B8] Elliot A. J. (1999). Approach and avoidance motivation and achievement goals. *Educ. Psychol.* 34 169–189. 10.1207/s15326985ep3403_3

[B9] Folkman S. Lazarus R. S. (1986). Stress-processes and depressive symptomatology. *J. Abnorm. Psychol.* 95 107–113. 10.1037//0021-843x.95.2.107 3711433

[B10] Gao W. Ping S. Liu X. (2020). Gender differences in depression, anxiety, and stress among college students: A longitudinal study from China. *J. Affect Disord* 263 292–300. 10.1016/j.jad.2019.11.121 31818792

[B11] Hammen C. (2005). Stress and depression. *Annu. Rev. Clin. Psychol.* 1 293–319. 10.1146/annurev.clinpsy.1.102803.143938 17716090

[B12] Harackiewicz J. M. Barron K. E. Pintrich P. R. Elliot A. J. Thrash T. M. (2002). Revision of achievement goal theory: Necessary and illuminating. *J. Educ. Psychol.* 94 638–645.

[B13] Holmes T. H. Rahe R. H. (1967). The social readjustment rating scale. *J. Psychosom. Res.* 11 213–218. 6059863 10.1016/0022-3999(67)90010-4

[B14] Hope V. Henderson M. (2014). Medical student depression, anxiety and distress outside North America: a systematic review. *Med. Educ.* 48 963–979. 10.1111/medu.12512 25200017

[B15] Hoyt M. A. Austenfeld J. Stanton A. L. (2016). Processing coping methods in expressive essays about stressful experiences: Predictors of health benefit. *J. Health Psychol.* 21 1183–1193. 10.1177/1359105314550347 25266296

[B16] Hu Lt Bentler P. M. (1999). Cutoff criteria for fit indexes in covariance structure analysis: Conventional criteria versus new alternatives. *Struct. Equ. Model. Multidisciplinar. J.* 6 1–55. 10.1080/10705519909540118

[B17] Jiang H. Li S. Yang J. (2018). Work Stress and Depressive Symptoms in Fishermen With a Smoking Habit: A Mediator Role of Nicotine Dependence and Possible Moderator Role of Expressive Suppression and Cognitive Reappraisal. *Front. Psychol.* 9:386–397. 10.3389/fpsyg.2018.00386 29632504 PMC5879124

[B18] Jiang L. Wang Y. Zhang Y. Li R. Wu H. Li C. (2019). The Reliability and Validity of the Center for Epidemiologic Studies Depression Scale (CES-D) for Chinese University Students. *Front. Psychiatr.* 10:315–326. 10.3389/fpsyt.2019.00315 31178764 PMC6537885

[B19] Keller A. Litzelman K. Wisk L. E. Maddox T. Cheng E. R. Creswell P. D. (2012). Does the perception that stress affects health matter? The association with health and mortality. *Health Psychol.* 31 677–684. 10.1037/a0026743 PMC3374921 22201278

[B20] Krys S. Otte K.-P. Knipfer K. (2020). Academic performance: A longitudinal study on the role of goal-directed rumination and psychological distress. *Anx. Stress Cop.* 2020 1–15. 10.1080/10615806.2020.1763141 32393058

[B21] Lazarus Richard S. (1966). Story telling and the measurement of motivation: the direct versus substitutive controversy. *J. Consult. Psychol.* 30 483–487. 5974528 10.1037/h0024008

[B22] Ling Y. He Y. Wei Y. Cen W. Zhou Q. Zhong M. (2016). Intrinsic and extrinsic goals as moderators of stress and depressive symptoms in Chinese undergraduate students: A multi-wave longitudinal study. *BMC Psychiatr.* 16:138–145. 10.1186/s12888-016-0842-5 27170095 PMC4864938

[B23] Liu H. Guo D. (2003). A research of the relationship between pretest anxiety, achievement goal orientation and test performance (in Chinese). *Psychol. Dev. Educ.* 19 64–68.

[B24] Lloyd C. Gartrell N. K. (1984). Psychiatric symptoms in medical students. *Comprehen. Psychiatr.* 25 552–565. 10.1016/0010-440x(84)90036-1 6509959

[B25] Meece J. L. Anderman E. M. Anderman L. H. (2006). Classroom goal structure, student motivation, and academic achievement. *Annu. Rev. Psychol.* 57 487–503. 10.1146/annurev.psych.56.091103.070258 16318604

[B26] Ntoumanis, Nikos, Vazou and Spiridoula. (2005). Peer Motivational Climate in Youth Sport: Measurement Development and Validation. *J. Sport Exercise Psy.* 27 432–455. 10.1123/jsep.27.4.432

[B27] Park H. Lee K.-S. (2020). The association of family structure with health behavior, mental health, and perceived academic achievement among adolescents: a 2018 Korean nationally representative survey. *BMC Public Health* 20:08655–z. 10.1186/s12889-020-08655-z PMC7164151 32299419

[B28] Puthran R. Zhang M. W. Tam W. W. Ho R. C. (2016). Prevalence of depression amongst medical students: a meta-analysis. *Med. Educ.* 50 456–468. 10.1111/medu.12962 26995484

[B29] Radloff L. S. (1977). The CES-D Scale A Self-Report Depression Scale for Research in the General Population. *Appl. Psychol. Measure.* 1 385–401.

[B30] Raykov T. Marcoulides A. G. (2007). Equivalent Structural Equation Models: A Challenge and Responsibility. *Struct. Equ. Model.* 14 695–700. 10.1080/10705510701303798

[B31] Rotenstein L. S. Ramos M. A. Torre M. Segal J. B. Peluso M. J. Guille C. (2016). Prevalence of Depression, Depressive Symptoms, and Suicidal Ideation Among Medical Students: A Systematic Review and Meta-Analysis. *JAMA* 316 2214–2236. 10.1001/jama.2016.17324 27923088 PMC5613659

[B32] Rothbaum F. Morling B. Rusk N. (2009). How Goals and Beliefs Lead People into and Out of Depression. *Rev. Gen. Psychol.* 13 302–314. 10.1037/a0017140

[B33] Ryder A. G. Yang J. Zhu X. Yao S. Yi J. Heine S. J. (2008). The cultural shaping of depression: somatic symptoms in China, psychological symptoms in North America? *J. Abnorm. Psychol.* 117 300–313. 10.1037/0021-843X.117.2.300 18489206

[B34] Ryu E. (2011). Effects of skewness and kurtosis on normal-theory based maximum likelihood test statistic in multilevel structural equation modeling. *Behav. Res. Methods* 43 1066–1074. 21671139 10.3758/s13428-011-0115-7

[B35] Shamsuddin K. Fadzil F. Ismail W. S. Shah S. A. Omar K. Muhammad N. A. (2013). Correlates of depression, anxiety and stress among Malaysian university students. *Asian J. Psychiatr.* 6 318–323. 10.1016/j.ajp.2013.01.014 23810140

[B36] Shen Y. Zhang Y. Chan B. S. M. Meng F. Yang T. Luo X. (2020). Association of ADHD symptoms, depression and suicidal behaviors with anxiety in Chinese medical college students. *BMC Psychiatr.* 20:180. 10.1186/s12888-020-02555-7 PMC7175542 32321462

[B37] Shrout P. E. Bolger N. (2002). Mediation in experimental and nonexperimental studies: new procedures and recommendations. *Psychol. Methods* 7 422–445. 10.1037/1082-989X.7.4.422 12530702

[B38] Sideridis G. D. (2007). Why are students with LD depressed? A goal orientation model of depression vulnerability. *J. Learn. Disab.* 40 526–539. 10.1177/00222194070400060401 18064978

[B39] Steiner-Hofbauer V. Holzinger A. (2020). How to Cope with the Challenges of Medical Education? Stress, Depression, and Coping in Undergraduate Medical Students. *Acad. Psychiatr.* 44 380–387. 10.1007/s40596-020-01193-1 32080825 PMC7359127

[B40] Stewart S. M. Lam T. H. Betson C. L. Wong C. M. Wong A. M. (1999). A prospective analysis of stress and academic performance in the first two years of medical school. *Med. Educ.* 33 243–250. 10.1046/j.1365-2923.1999.00294.x 10336754

[B41] Street H. (2002). Exploring Relationships Between Goal Setting, Goal Pursuit and Depression: A Review. *Austr. Psychol.* 37 95–103. 10.1080/00050060210001706736

[B42] Tian L. Yu T. Huebner E. S. (2017). Achievement Goal Orientations and Adolescents’ Subjective Well-Being in School: The Mediating Roles of Academic Social Comparison Directions. *Front. Psychol.* 8:37–47. 10.3389/fpsyg.2017.00037 28197109 PMC5281619

[B43] Tuominen-Soini H. Salmela-Aro K. Niemivirta M. (2008). Achievement goal orientations and subjective well-being: A person-centred analysis. *Learn Instr.* 18 251–266. 10.1016/j.learninstruc.2007.05.003

[B44] Ursin H. Eriksen H. (2007). Cognitive activation theory of stress, sensitization, and common health complaints. *Ann. N. Y. Acad. Sci.* 1113 304–310. 10.1196/annals.1391.024 17584977

[B45] Valle A. Cabanach R. G. Nunez J. C. Gonzalez-Pienda J. Rodriguez S. Pineiro I. (2003). Multiple goals, motivation and academic learning. *Br. J. Educ. Psychol.* 73(Pt 1), 71–87. 10.1348/000709903762869923 12639278

[B46] van Dam A. Noordzij G. Born M. (2020). Thriving Under Uncertainty: The Effect of Achievement Goal Orientation on Job Insecurity and Flourishing. *Soc. Indic. Res.* 3 2337–2334. 10.1007/s11205-020-02337-4

[B47] Vandewalle D. Nerstad C. G. L. Dysvik A. (2019). Goal Orientation: A Review of the Miles Traveled and the Miles to Go. *Annu. Rev. Organ. Psych.* 6 115–144. 10.1146/annurev-orgpsych-041015-062547

[B48] Vergara C. Roberts J. E. (2011). Motivation and goal orientation in vulnerability to depression. *Cogn. Emot.* 25 1281–1290. 10.1080/02699931.2010.542743 21432630

[B49] Weiner B. (1985). An attributional theory of achievement motivation and emotion. *Psychol. Rev.* 92 548–573. 10.1037/0033-295X.92.4.548 3903815

[B50] Wooley S. C. Blackwell B. Winget C. (1978). A learning theory model of chronic illness behavior: theory, treatment, and research. *Psychosom. Med.* 40 379–401. 10.1097/00006842-197808000-00003 715141

[B51] Yang Q. Tian L. Huebner E. S. Zhu X. (2019). Relations among academic achievement, self-esteem, and subjective well-being in school among elementary school students: A longitudinal mediation model. *Sch. Psychol.* 34 328–340. 10.1037/spq0000292 30474992

[B52] Yusoff M. S. Abdul Rahim A. F. Baba A. A. Ismail S. B. Mat, Pa M. N. (2013). Prevalence and associated factors of stress, anxiety and depression among prospective medical students. *Asian J. Psychiatr.* 6 128–133. 10.1016/j.ajp.2012.09.012 23466109

[B53] Zhang J. Wu Z. Fang G. Li J. Han B.-X. Chen Z.-Y. (2010). Development of the Chinese age norms of CES-D in urban area (in Chinese). *Chin. Ment. Health J.* 24 139–143. 10.3969/j.issn.1000-6729.2010.02.015

