# Supplementary material for: The Mediating Role of Stress Perception in Pathways Linking Achievement Goal Orientation and Depression in Chinese Medical Students
Source: Front Psychol. 2021 Feb 19;12:614787. doi: 10.3389/fpsyg.2021.614787 (PMC7934623; doi:10.3389/fpsyg.2021.614787)
Supplement: Supplementary file 1 [file Data_Sheet_1.PDF]

Appendix A. Stress Perception Scale

Please select the answer that best fits you and tick the appropriate option. Please read each question carefully before answering. There is no right or wrong answer. Thanks for your cooperation!

1. During the past three months, would you say that you experienced	a lot of stress<br>☐5	a mild amount stress<br>☐4	a moderate amount of stress<br>☐3	relatively little stress<br>☐2	almost no stress at all<br>☐1
2. What role does stress play in your daily learning and life?	only has positive contributions, the pressure that keeps motives going<br>☐5	more positive contributions<br>☐4	no perception<br>☐3	negative effect, affects mood<br>☐2	totally negative, making me uneasy<br>☐1
3. Who do you ask for help first to reduce stress in your life?	family member or lovers<br>☐5	friends<br>☐4	teachers<br>☐3	mental health counselors<br>☐2	all on my own<br>☐1
	strongly fit me	somewhat fit me	uncertain	somewhat does not fit me	strongly does not fit me
4. The lectures are difficult, I am under great academic pressure and worring about failing in the final examination.	☐5	☐4	☐3	☐2	☐1
5. The pressure of competition among schoolfellows makes it difficult to get a scholarship.	☐5	☐4	☐3	☐2	☐1
6. CET-4, CET-6, computer grade examination, as well as all kinds of certificate examination are very difficult.	☐5	☐4	☐3	☐2	☐1
7. I'm upset for long-term personal career planning of choice issues (such as major, study for the graduate degree, abroad or employment, etc.)	☐5	☐4	☐3	☐2	☐1
8. Association work, part-time jobs, or other extracurricular activities often get in my way.	☐5	☐4	☐3	☐2	☐1
9. I have encountered affection problems and I'm struggling with them.	☐5	☐4	☐3	☐2	☐1
10. My family has very high expectations for me, and they want me to obtain great success.	☐5	☐4	☐3	☐2	☐1
11. The disharmony of the family relationship is uncomfortable for me.	☐5	☐4	☐3	☐2	☐1
