# Supplementary material for: The Mediating Role of Stress Perception in Pathways Linking Achievement Goal Orientation and Depression in Chinese Medical Students
Source: Front Psychol. 2021 Feb 19;12:614787. doi: 10.3389/fpsyg.2021.614787 (PMC7934623; doi:10.3389/fpsyg.2021.614787)
Supplement: Supplementary file 3 [file Table_2.DOCX]

Supplementary Table 1. Factor loadings for Stress Perception scale V2.0

| Item | Rotated Component Matrix | |
| --- | --- | --- |
|  | Perceived Stress | Stress Related Cognition |
| SP7 | **0.808** | 0.064 |
| SP13 | **0.738** | 0.154 |
| SP4 | **0.671** | -0.079 |
| SP10 | **0.661** | 0.221 |
| SP6 | **0.657** | 0.003 |
| SP5 | **0.589** | -0.142 |
| SP9 | **0.567** | -0.129 |
| SP12 | **0.525** | 0.337 |
| SP14 | -0.024 | -0.013 |
| SP2 | -0.062 | **0.827** |
| SP3 | 0.083 | **0.797** |
| SP1 | 0.120 | **0.751** |
| SP11 | 0.149 | -0.430 |
| SP8 | 0.226 | 0.357 |

Extraction Method: Principal Component Analysis.

Rotation Method: Varimax with Kaiser Normalization.

a. Rotation converged in 3 iterations.

Supplementary Table 2. Reliability of Stress Perception scale V2.0

| Stress Perception scale | Cronbach’s alpha | Composite Reliability | Average Variance Extracted |
| --- | --- | --- | --- |
| Total | 0.78 |  |  |
| Perceived Stress (PS) | 0.812 | 0.857 | 0.432 |
| Stress Related Cognition (SRC) | 0.779 | 0.856 | 0.665 |

Supplementary Table 3. Composite reliability coefficients and average variance extracted for the variables for three scales

| Scale | Composite Reliability | Average Variance Extracted |
| --- | --- | --- |
| Achievement Goal Orientation scale |  |  |
| Mastery-Approach (MAP) | 0.82 | 0.347 |
| Performance-Approach (PAP) | 0.822 | 0.347 |
| Mastery-Avoidance (MAV) | 0.768 | 0.399 |
| Performance-Avoidance (PAV) | 0.827 | 0.444 |
| Stress Perception scale |  |  |
| Perceived Stress (PS) | 0.84 | 0.431 |
| Stress Related Cognition (SRC) | 0.783 | 0.546 |
| Center for Epidemiological Studies-Depression scale |  |  |
| Somatic concern (SC) | 0.803 | 0.323 |
| Depressive affect (DA) | 0.789 | 0.354 |
| Anhedonia (AN) | 0.783 | 0.478 |

Supplementary Table 4. Factor loadings for stress perception scale

| Item | Rotated Component Matrix | |
| --- | --- | --- |
|  | Perceived Stress | Stress Related Cognition |
| SP5 | **0.739** | -0.239 |
| SP4 | **0.713** | -0.227 |
| SP6 | **0.681** | -0.272 |
| SP9 | **0.641** | -0.233 |
| SP10 | **0.631** | -0.022 |
| SP13 | **0.623** | -0.083 |
| SP12 | **0.548** | 0.416 |
| SP7 | **0.490** | -0.390 |
| SP3 | -0.089 | **0.779** |
| SP1 | -0.171 | **0.733** |
| SP2 | -0.258 | **0.703** |

Extraction Method: Principal Component Analysis.

Rotation Method: Varimax with Kaiser Normalization.

a. Rotation converged in 3 iterations.

Supplementary Table 5. Factor loadings for achievement goal orientations scale

| Item | Rotated Component Matrix | | | |
| --- | --- | --- | --- | --- |
|  | Mastery-Approach | Performance-Approach | Performance-Avoidance | Mastery-Avoidance |
| AGO7 | **0.712** | 0.095 | -0.044 | 0.158 |
| AGO1 | **0.699** | 0.022 | -0.055 | 0.032 |
| AGO17 | **0.667** | -0.010 | 0.040 | -0.130 |
| AGO22 | **0.623** | 0.069 | -0.153 | 0.027 |
| AGO10 | **0.609** | 0.038 | -0.015 | 0.181 |
| AGO14 | **0.591** | 0.154 | -0.128 | 0.142 |
| AGO25 | **0.569** | 0.175 | -0.030 | -0.012 |
| AGO19 | **0.376** | 0.174 | 0.287 | -0.211 |
| AGO5 | **0.332** | 0.211 | -0.102 | 0.311 |
| AGO12 | -0.001 | **0.711** | 0.036 | 0.176 |
| AGO13 | 0.292 | **0.656** | 0.041 | 0.139 |
| AGO24 | 0.003 | **0.655** | 0.302 | -0.022 |
| AGO3 | 0.060 | **0.639** | -0.048 | 0.164 |
| AGO18 | 0.079 | **0.632** | -0.072 | -0.030 |
| AGO26 | 0.286 | **0.584** | 0.131 | 0.037 |
| AGO6 | 0.390 | **0.522** | 0.059 | -0.017 |
| AGO9 | 0.414 | **0.437** | -0.029 | 0.155 |
| AGO29 | -0.103 | **0.385** | 0.259 | 0.098 |
| AGO21 | -0.027 | -0.020 | **0.705** | 0.006 |
| AGO8 | 0.038 | 0.107 | **0.700** | 0.186 |
| AGO15 | -0.042 | 0.099 | **0.700** | 0.156 |
| AGO16 | -0.146 | -0.012 | **0.668** | 0.085 |
| AGO2 | 0.032 | 0.100 | **0.641** | 0.161 |
| AGO28 | -0.162 | 0.040 | **0.575** | 0.196 |
| AGO23 | -0.026 | 0.238 | 0.018 | **0.687** |
| AGO11 | 0.111 | 0.033 | 0.183 | **0.648** |
| AGO4 | 0.023 | 0.108 | 0.223 | **0.638** |
| AGO20 | 0.248 | 0.034 | 0.159 | **0.595** |
| AGO27 | -0.023 | 0.032 | 0.277 | **0.584** |

Extraction Method: Principal Component Analysis.

Rotation Method: Varimax with Kaiser Normalization.

a. Rotation converged in 6 iterations.

Supplementary Table 6. Factor loadings for CES-D scale

| Item | Rotated Component Matrix | | |
| --- | --- | --- | --- |
|  | Somatic Concern | Depressive Affect | Anhedonia |
| CESD6 | **0.734** | 0.269 | 0.156 |
| CESD1 | **0.678** | 0.218 | 0.005 |
| CESD5 | **0.648** | 0.112 | 0.077 |
| CESD7 | **0.580** | 0.320 | 0.287 |
| CESD18 | **0.557** | 0.519 | 0.096 |
| CESD3 | **0.550** | 0.382 | 0.169 |
| CESD11 | **0.496** | 0.062 | 0.147 |
| CESD9 | **0.443** | 0.387 | 0.413 |
| CESD2 | **0.305** | 0.141 | 0.052 |
| CESD19 | **0.302** | 0.724 | 0.135 |
| CESD17 | 0.009 | **0.707** | 0.052 |
| CESD15 | 0.200 | **0.631** | 0.251 |
| CESD10 | 0.393 | **0.554** | 0.162 |
| CESD20 | 0.359 | **0.525** | 0.170 |
| CESD14 | 0.450 | **0.514** | 0.136 |
| CESD13 | 0.293 | **0.456** | 0.210 |
| CESD8 | 0.189 | 0.071 | **0.759** |
| CESD16 | 0.235 | 0.140 | **0.730** |
| CESD12 | 0.297 | 0.155 | **0.702** |
| CESD4 | -0.188 | 0.220 | **0.556** |

Extraction Method: Principal Component Analysis.

Rotation Method: Varimax with Kaiser Normalization.

a. Rotation converged in 5 iterations.
